# Integrating Biobehavioral and Environmental Components of Developmental Psychopathology via Interpersonal Dynamics: An RDoC-Advancing Model

**DOI:** 10.1007/s10802-023-01110-8

**Published:** 2023-08-21

**Authors:** Jennifer A. Somers, Tiffany C. Ho, Danielle Roubinov, Steve S. Lee

**Affiliations:** 1 Department of Psychology, University of California, 502 Portola Plaza, Pritzker Hall, CA 6658 Los Angeles, USA; 2 Department of Psychiatry, University of North Carolina, Chapel Hill, NC USA; 3 Department of Psychiatry and Behavioral Sciences, University of California, San Francisco, CA USA

**Keywords:** Caregiver-child dynamics, Synchrony, RDoC, Dynamic structural equation model, Respiratory sinus arrhythmia

## Abstract

Although the Research Diagnostic Criteria (RDoC) framework proposes biological and environmental mechanisms intersect in the etiology of psychopathology, there is no guidance on how to define or measure experiences in the environment *within* the RDoC matrix. Interpersonal dynamics during caregiver–child interactions involve temporal coordination of interacting partners’ biobehavioral functioning; repeated experiences of signaling to caregivers and responding to caregivers’ signals shape children’s subsequent socioemotional and brain development. We begin with a review of the extant literature on caregiver-child dynamics, which reveals that RDoC’s units of analysis (brain circuits, physiology, behavior, and self-report) are inextricably linked with moment-to-moment changes in the caregiving environment. We then offer a proof-of-concept for integrating biobehavioral RDoC units and environmental components via caregiver-child dynamics. Our approach uses dynamic structural equation models to estimate within-dyad dynamics involving arousal, social, cognitive, and negative or positive affective processes based on second-by-second changes in parasympathetic activity (RSA) during a conflict discussion and a positive event-planning task. Our results illustrate variation in parent–child RSA synchrony, suggesting differences depending on the driver (i.e., child- or parent-led) and on the unique and intersecting domains involved (e.g., positive or negative affect valence systems). We conclude with recommendations for conducting robust, methodologically rigorous studies of interpersonal dynamics that advance the RDoC framework and provide a summary of the clinical implications of this research. Examining caregiver-child dynamics during and across multiple dyadic interaction paradigms that differentially elicit key domains of functioning can deepen understanding of how caregiver- and child-led interpersonal dynamics contribute to child psychopathology risk.

**Supplementary Information:**

The online version contains supplementary material available at 10.1007/s10802-023-01110-8.

Traditional diagnostic categories from the Diagnostic and Statistical Manual of Mental Disorders (DSM) and the International Classification of Disease (ICD) suffer from etiologic heterogeneity, frequent comorbidity, and arbitrary cutoffs that delimit clinical utility. These limitations prevent the translation of basic science knowledge to reduce the considerable burden of mental illness (Cuthbert & Insel, 2013). Advancing an etiology-based taxonomy of psychopathology, the NIMH Research Domain Criteria (RDoC) initiative launched an experimental framework for researchers to use to identify reliable and valid psychobiological mechanisms that underlie psychiatric symptoms (Cuthbert, 2014). Following this conceptualization of psychopathology as alterations in biological and psychological processes across key domains of functioning, the major RDoC framework is a two-dimensional matrix of superordinate domains whose constructs can be assayed with separable units of analysis (e.g., genes, molecules, brain circuits, physiology, behavior, self-report) and assessment paradigms. By elucidating neurodevelopmental *processes,* RDoC aims to achieve the highest level of construct validity (Morris & Cuthbert, 2012). In turn, identification of etiological processes may spur long-awaited innovations in assessment, prevention, and intervention efforts (Cuthbert & Insel, 2013).

RDoC has already led to changes in the conceptualization of adult psychopathology, including examination of brain-behavior dimensions of transdiagnostic constructs and treatment outcomes (Sanislow, 2020). Yet, RDoC has yet to fulfill its promises for child and adolescent mental health, a noteworthy shortfall in the context of the nation’s worsening crisis in child and adolescent psychopathology (U.S. Surgeon General’s Advisory, 2021; CDC, 2011–2021). Although the RDoC initiative has a mandate to integrate “the fundamental genetic, neurobiological, behavioral, *environmental*, and experiential components that comprise mental disorders” (NIMH, 2008, emphasis added), it remains largely agnostic with respect to how the environment should be defined or measured, including intersections with components within the matrix (Conradt et al., 2021; Mittal & Wakschlag, 2017). Early experiences with caregivers are an especially important source of environmental input that directly affect child neurodevelopment (King et al., 2021), as evident across multiple domains (e.g., social processes, cognitive systems, regulatory systems; Feldman, 2012; Sroufe, 2005; Zeanah et al., 2017). Consistent with recent calls to improve integration of environmental influences with constructs within the RDoC framework (Beauchaine & Hinshaw, 2020; Conradt et al., 2021; Mittal & Wakschlag, 2017), we contend that critical intersections between the external caregiving environment and the developing child constitute the most potent risk factors for psychopathology. King et al. (2021) offer an important first step in advancing this research agenda in their conceptualization of the caregiving environment *within* the social processes domain of the existing RDoC matrix. We laud their explication of how the caregiving environment, though assessed extrinsically to the focal child, is critical to child neurodevelopment. At the same time, highly influential transactional and bioecological models contend that *both* dyad members actively shape the caregiver-child relationship (Bronfenbrenner & Morris, 2006; Feldman, 2007, 2012; Sameroff, 2009) and discourage reducing dynamic relationship influences to any one individual (Bornstein, 2013).

In this paper, we first define interpersonal dynamics and review theoretical and empirical support for examining caregiver-child dynamics in moment-to-moment indices across separable brain circuits, physiology, behavior, and self-report units. Then, we offer a methodological proof-of-concept of our novel *interpersonal* approach for assessing intersections between the caregiving environment and biobehavioral components within the RDoC matrix vis-à-vis caregiver-child interpersonal dynamics. The review concludes with an agenda for future RDoC-advancing research and clinical implications of caregiver-child interpersonal dynamics for assessing, preventing, and mitigating child psychopathology risk.

## Defining Caregiver-Child Dynamics

Harmonizing the jingle-jangle of conceptual terms in the literature (e.g., “synchrony,” “attunement,” “concordance”), we operationalize caregiver-child “interpersonal dynamics” as the temporal coordination in the ebb and flow of parents’ and their children’s biology, behavior, and experience during social interactions (Butler, 2011). Caregiver-child dynamics are tied to well-validated conceptual frameworks, including dynamic systems perspectives on emotions and developmental psychopathology (e.g., Butler, 2011; Granic, 2005), parent–child emotion regulation dynamics (Morris et al., 2018; Ratliff et al., 2022), and attachment-based biobehavioral regulatory models (Feldman, 2007, 2012). These frameworks share several central tenets. First, interpersonal dynamics consist of whole-body processes that involve the interpersonal maintenance of physiological and behavioral homeostasis (Butler, 2011). Second, caregiver-child dynamics typically follow a normative developmental progression. Biological preparedness for interpersonal dynamics is evident in utero (Feldman, 2007) and, after infancy, the emergence of language and symbolic thought may allow for more complex forms of synchrony than those focused on simple behavioral changes, including synchrony in attention, thoughts, intentions, goals, and experiences (Bell, 2020; Feldman, 2007; Harrist & Waugh, 2002). Finally, through repeated experiences, caregiver-child dynamics shape the evolving dyadic relationship and the future individual development of both members of the dyad (King et al., 2021; Leclère et al., 2014; Perlman et al., 2022).

## Evidence of Caregiver-Child Dynamics in RDoC Units

In this section, we synthesize evidence across theoretical, narrative, systematic, and meta-analytic reviews demonstrating that within-dyad caregiver-child dynamics are evident in well-established neural, physiological, behavioral, and experiential units of analysis. Under the broader umbrella of “interpersonal dynamics,” “positive synchrony” refers to reciprocated *in-phase* changes that are matched in the direction of change in their partner. In contrast, “negative synchrony” refers to *anti-phase* changes in one individual that are inversely associated with changes in their partner. We primarily focus on synchrony, although there are numerous operationalizations of interpersonal dynamics, including dyad-level assessment (e.g., dyadic flexibility; Grumi et al., 2022; Hollenstein et al., 2013; the dyad’s tendency to move in and out of synchrony; Mayo & Gordon, 2020).

Table 1 highlights the domains (superordinate constructs) for which there is empirical evidence of caregiver-child dynamics among typical developmental, at-risk, and clinical populations, organized by the units of analysis in the RDoC matrix and key paradigms. Caregiver-child dynamics have been reliably established using multiple assessment paradigms, including unstructured play tasks and standardized stress and stress and recovery tasks (Atkinson et al., 2016; Golds et al., 2022; Leclère et al., 2014; Miller et al., 2023; Provenzi et al., 2018). Notably, units of analysis and assessment paradigms are orthogonal to the domains, such that each construct in the matrix can be assessed in various measurement classes and assessment paradigms (Cuthbert, 2014).

**Table 1:** Reviews of units of analysis and paradigms for assessing caregiver-child interpersonal dynamics in RDoC constructs

Units of Analysis					
Domains	Circuits	Physiology	Behavior	Self-report	Paradigms
Negative valence systems	prefrontal cortex^a,b^; dorsolateral prefrontal cortex^b,c^	ANS^a,b,d,e^^;^ PNS^f^; HR^b,d,e^; HPA, dysregulated HPA axis, average cortisol levels^b,d,e,g,h^; skin conductance^b,e^	crying, sadness, worry^a,d,i^; facial expressions^a,d,i,j,k,s^; increased conflict detection, physical and relational aggression^d,j,l,m^; approach, avoidance, withdrawal^i,n,o^	self-reported distress^p^	labtab frustrative nonreward^b,h^; fear film^f^
Positive valence systems	prefrontal cortex^a,b^	HR change^a,b^		PANAS^p^	
Cognitive	prefrontal cortex^a,b^; oscillations (scalp eeg)^a^; dorsolateral prefrontal and frontopolar cortex^b,c^	HR deceleration^a,b,e^	coherent discourse, coherent sentences^j^; off-task behaviors^j,^^l,m^		
Social processes	prefrontal cortex^a,b^; superior temporal sulcus^b,q^; V1-FFA-STS-amygdala & V1-FFA-STS-VS ^b,q^	Sympathetic activity^b,d,e,g^; HPA axis activation and down-regulation^b,d,e,g^, vagal tone, vagal withdrawal^a,b,f^; HR/bp/respiration, HR variability^a,b,d,e^; skin temperature^b,e^; skin conductance, skin conductance response ^b,e^	reciprocal eye contact, gaze following, eye gaze detection, eye gaze aversion/contact, joint attention^a,i,j,n,o^; behavior observation/coding systems, facial affect production, developmentally appropriate perception of one’s emotional states, mimicry; imitation of facial gestures; reciprocal emotional expression^a,d,i,j,k,n,o,s^; vocalizations^a,i,k^; interactive play^o,i,n^; distress upon separation^i^; gestural/postural expressions^o^		multimodal social paradigms^b,e,f,g,h,i,k,r^
Arousal & regulatory systems	EEG theta rhythms^q^	Eeg^a,b,h^; HR^a,b,d,e^; galvanic skin response^b,e^	Affective states, emotional reactivity^a,d,i,j,k,n,o,s^		cardiac PEP^b^; HRV^a,b,d,f^; electrodermal responding^d,e^
Sensorimotor systems	somatosensory cortex^a^; dorsolateral prefrontal cortex^c^	Oscillatory rhythm^a^	Activity level^n,o^		

All citations refer to reviews that offer a synthesis of evidence of interpersonal caregiver-child dynamics for a given construct, except Henry et al. (2022) who offer preliminary evidence of subjective parent–child dynamics from a single study

*ANS* Autonomic nervous system, *EEG* Electroencephalogram, *HPA* Hypothalamus–pituitary–adrenal, *HR* Heart rate, *HRV* HR Variability, *IBI* Interbeat interval, *PEP* Preejection period, *PNS* Parasympathetic nervous system,

^a^Bell (2020)

^b^DePasquale (2020)

^c^Ratliff et al. (2022)

^d^Birk et al. (2022)

^e^Davis et al. (2018)

^f^Miller et al. (2023)

^g^Atkinson et al. (2016)

^h^DiLorenzo et al. (2022)

^i^Leclère et al. (2014)

^j^Harrist and Waugh (2002)

^k^Provenzi et al. (2018)

^l^Patterson (1982)

^m^Patterson et al. (1992)

^n^Davis et al. (2017)

^o^Feldman et al. (2007)

^p^Henry et al. (2022)

^q^Turk et al. (2022)

^r^Golds et al. (2022)

^s^Grumi et al. (2022)

### Brain Circuits

Brain-to-brain synchronization in cortical regions is reported in caregiver-child interaction (Turk et al., 2022), often measured using “hyperscanning” (simultaneous recording of more than one brain) methods, including electroencephalogram (EEG), and functional near infrared spectroscopy (fNIRS) (Bell, 2020; Ratliff et al., 2022; Turk et al., 2022). EEG hyperscanning methods revealed mother-infant brain-to-brain synchrony at central and parietal scalp electrodes (Bell, 2020), synchrony in EEG theta rhythms (Turk et al., 2022), and coordination of EEG frontal asymmetry (Di Lorenzo et al., 2022). Studies using fNIRS have also demonstrated increased parent–child inter-brain synchrony in frontal regions, including portions of the prefrontal cortex and the frontropolar cortex, and more ventral regions found in the temporal cortex, such as the fusiform gyrus and the superior temporal sulcus (DePasquale, 2020; Turk et al., 2022).

### Physiology

Interpersonal dynamics are also evident in physiological systems, including the autonomic and endocrine systems, that coordinate with neural systems to affect the brain-body stress response (Davis et al., 2018; DePasquale, 2020). The strongest evidence of coordination of caregiver's and children’s physiological functioning has been assessed by cortisol, the end product of the hypothalamic–pituitary–adrenal (HPA) axis (Atkinson et al., 2016; Birk et al., 2022; Davis et al., 2018; Di Lorenzo et al., 2022). Interpersonal dynamics have also been reliably established in nonspecific autonomic measures, including heart rate and interbeat intervals (IBI) and thermal facial imprints (Davis et al., 2018; DePasquale, 2020), as well as within both the sympathetic (e.g., electrodermal activity/skin conductance, salivary alpha amylase, cardiac PEP) and parasympathetic branches (e.g., vagal withdrawal indexed by decreased respiratory sinus arrhythmia [RSA] or heart rate variability in the high frequency domain [HF-HRV]) of the autonomic nervous system (Atkinson et al., 2016; Birk et al., 2022; Davis et al., 2018; DePasquale, 2020). Recent meta-analytic work suggests that, on average, for mother–child dyads from infancy to adolescence, there generally is concurrent positive RSA synchrony (Miller et al., 2023).

### Behavior

Interpersonal dynamics have been established in gaze, eye contact, vocalizations, speech turn-taking, vocal affect, facial affect, gestures, postures, physical proximity, and tactile behaviors (e.g., affectionate touch) during face-to-face interactions; these interpersonal dynamics are evident as early as 3 months of age (Bell, 2020; Feldman, 2012; Feldman et al., 2007; Golds et al., 2022; Grumi et al., 2022; Harrist & Waugh, 2002; Leclère et al., 2014) and in diverse cultural contexts (Bornstein, 2013). Most assessments focus on observer coding of facial and vocal affect and social engagement (e.g., gestures, body language) from videorecorded interactions, but assessment (e.g., of the language environment, physical proximity) can also be automated through wearable technology (e.g., King et al., 2021) and other computerized assessment methods, which may spur more nuanced understanding of specific interpersonal behavioral dynamics.

### Self-Report

Interpersonal coordination of subjective experience (e.g., shared intentionality) has historically been inferred from caregiver and child cooperation on tasks with shared goals, such as puzzle tasks or imitation games (Bell, 2020; Feldman, 2007). However, the well-established video-mediated recall paradigm (VMR; Lorber, 2007; Welsh & Dickson, 2005) asks participants to watch immediately preceding videorecorded interactions and report their feelings, distress, thoughts, perceptions, or self-statements. Interpersonal dynamics in subjective experience between caregivers and children as young as 7 years of age have been reliably assessed (Welsh & Dickson, 2005). One study leveraged dyadic VMR to examine parent–child interpersonal dynamics in self-reported emotional experience where positive synchrony in parents’ and adolescents’ recalled affect followed a conflict discussion (Henry et al., 2022).

## Proof-of-Concept Study: Examining Parent–Child Coordination of Parasympathetic Nervous System Functioning During Conflict and Positive Event-Planning

Conceptualizing caregiver–child interactions as the product of interpersonal dynamics using RDoC units affords the opportunity to examine *real-time intersections* between children’s most salient environmental context – the caregiver-child relationship – and intrapersonal biobehavioral processes that unfold from one moment to the next. Our proof-of-concept study aims to motivate future research that prioritizes: (a) moment-to-moment assays to uncover caregiver-child dynamics, including the direction in which state-like fluctuations in caregiver’s and youth’s biobehavioral processes impact or are impacted by each other and (b) pinpoint within-dyad differences in dynamics across RDoC domains that clarify between-child differences in risk for psychopathology. We assessed parent–child synchrony involving vagal functioning (indexed by respiratory sinus arrhythmia [RSA], a measure of parasympathetically-mediated vagal influences on cardiometabolic output) in different interpersonal assessment paradigms, among a sample of emotionally at-risk preadolescent girls and their parents. Specifically, we evaluated within-dyad, parent- and child-led RSA synchrony in two ecologically valid interaction contexts (i.e., conflict discussion and positive event-planning) that each recruit processes arousal, social, and cognitive RDoC domains, but differentially elicit negative and positive valence systems, respectively. To exemplify the proposed approach, our primary aims were to evaluate variation in the presence of within-dyad parent- and child-led RSA synchrony in (a) a conflict discussion task, (b) a pleasant event discussion, and (c) to explore whether families evaluated different patterns of synchrony in these tasks.

## Method

### Participants

The sample for our methodological demonstration included caregiver-daughter dyads participating in an ongoing investigation of the cognitive, familial, and psychophysiological correlates of emotional development in 6- to 11-year-old girls, the Developmental Research on Emotion and Mental Health in Girls (DREAMING) Study. Consistent with principles of RDoC, targeted recruitment efforts ensured sufficient range in girls’ negative emotionality without regard to a specific diagnostic taxon. Middle childhood is a critical inflection point for changing interpersonal, emotional, and physiological dynamics. We prioritized examination of parent-daughter dynamics in particular given girls’ unique vulnerability to developing internalizing problems, especially among dyads with girls who are temperamentally at-risk for emotional problems. This prescribed developmental period also temporally precedes adolescence, which is witness to acute increases in internalizing problems. Eligibility criteria included: 1) child who identifies as female between 6 and 11 years old during the time of screening, 2) fluency in English, 3) child must not have a history of seizures or seizure disorder and 4) child must not have been previously diagnosed with intellectual disability or an autism spectrum disorder (ASD). The University of California, Los Angeles IRB approved all study procedures prior to recruitment or data collection.

Data collection for the DREAMING Study was halted by the COVID-19 pandemic and recently resumed; data collection for the proof-of-concept subsample took place between March 2019 and February 2020. The sample who participated in a conflict discussion task or positive event-planning task included 28 girls between 6 and 11 years of age (*M*_age_ = 8.54 years, *SD* = 1.82 years) and their caregivers (92.9% female). Children and families had a racial/ethnic distribution that is representative of the local geographic area. The children were ethnically diverse; most children were identified by their caregivers as multiracial (55.7%). All caregivers were the child’s biological parent (85.7% female; 14.3% male); most caregivers were married (82.1%) and had attained a bachelor’s degree or above (89.3%). The modal average annual income of the sample was $125,000–$150,000, with a wide range from less than $25,000 to over $300,000 per year, for an average household size of 3.64 (SD = 1.06).

### Recruitment

Participants were recruited for the parent study from community settings, including pediatric offices, mental health service providers, tutoring centers, community/recreation centers, and local schools. Interested families contacted the research team and were carefully screened using a standardized script. If they satisfied eligibility criteria and they remained interested in the study, families were then scheduled for a laboratory visit.

### Procedures

At the laboratory visit, parents provided informed consent and children assented to study procedures; parents and daughters then completed rating scales, clinical interviews, behavioral paradigms, and laboratory-based parent–child interaction tasks. During the parent–child interaction tasks, parent-daughter dyads completed two 5-min interaction tasks in a fixed order: a Conflict Discussion followed by a Positive Event-Planning Task. Prior to the discussion tasks, parents were asked to rate common sources of conflict, using the Issues Checklist (Prinz et al., 1979), and commonly enjoyed activities, using the Pleasant Events Checklist (MacPhillamy & Lewinsohn, 1982). For the Conflict Discussion, an experimenter selected a source of conflict that the parent rated highly and indicated was unresolved, and then asked them to discuss it for 5 min. Similarly, in the Positive Event-Planning Task, an experimenter selected a pleasant activity that the parent rated highly, and then asked them to spend 5 min planning the pleasant event. The Conflict Discussion and Positive Event-Planning Tasks have been shown to differentially elicit negative and positive negative behaviors, respectively (Richmond et al., 2020). Participants were compensated $100 for the 4-h laboratory visit.

### Measures

#### Parent and Daughter Respiratory Sinus Arrhythmia (RSA)

Disposable Ag/AgCl electrodes were placed on each participants' chest in a modified Lead II placement, on the right clavicle, left clavicle, and lower right rib cage. Electrocardiogram (ECG) data were acquired using the Biopac MP160 system (Biopac Systems Inc., Goleta, CA) at a sampling rate of 2000 Hz. Coders used Acqknowledge Version 5.0 (Biopac Systems Inc., Goleta, CA) to process the data, manually correct misidentified or unidentified R-spikes, such as ectopic beats due to physical movement, and to obtain interbeat interval (IBI) data.

We estimated time-varying RSA for each five-minute discussion task using the MATLAB toolbox RSASeconds (Gates et al., 2015). Each of the cleaned IBI series was interpolated at 4 Hz using a cubic spline to create equal data intervals. The data were then tapered using Peak Matched Multiple Windows (PM MW) and a short-time Fourier transform (STFT) was applied to moving 32-s IBI windows in order to obtain an estimate of the power spectrum for the 16th second of the window. Power estimates were obtained within the adult respiration frequency band (0.12–0.40 Hz; Berntson, Quigley, & Lozano, 2007) for caregiver participants and within age-appropriate frequency bands for child participants (Shader et al., 2018). In short, the combination of PM MW and STFT produces point estimates of time-varying RSA for the central second in every rolling 32-s window, while drawing on information from the 16 s before and after the central second (Gates et al., 2015); notably, this focal tapering method has been shown to capture changes in RSA without requiring first-differencing (Gatzke-Kopp et al., 2022). The PM MW/STFT method has been validated among adult and caregiver-child dyads (Gatzke-Kopp et al., 2022; Somers et al., 2021; Zhang et al., 2022).

#### Data Analytic Plan: Assessment of RSA Synchrony

Idiographic, single-level dynamic structural equation models (DSEMs; Asparouhov et al., 2018) were tested using M*plus* Version 8.3 (Muthén & Muthén; 1998–2017) to evaluate within-dyad time-lagged RSA synchrony (defined in terms of how one partner’s RSA responsivity influences their own and their partner’s RSA responsivity during the subsequent second, after accounting for intrapersonal stability in RSA responsivity) for each dyad, per task. M*plus* uses Bayesian Markov Chain Monte Carlo (MCMC) with a Gibbs Sampler to estimate DSEMs. We used two unthinned chains, each running for a maximum of 100,000 iterations to ensure the estimation was stable. We allowed the algorithm to terminate prematurely if the Potential Scale Reduction factor dropped below 1.05 (Gelman & Rubin, 1992). We used the default prior distributions in M*plus*. Posterior distributions were summarized with the median.

Lagged variables of daughters’ and parents’ RSA were created in M*plus* and were latent mean centered to disaggregate within-person fluctuations from trait-like RSA levels (Hamaker & Grasman, 2015). In each single-level DSEM, the within-dyad intercepts, intercept variances and covariance, and all within-person stability (autoregressive paths) and within-dyad RSA synchrony (cross-lagged regression paths in daughters’ and parents’ RSA) were estimated. Within-dyad parent-driven RSA synchrony was operationalized as the effect of fluctuations in a parents’ RSA at one time point on fluctuations in their daughters’ RSA the subsequent second, adjusting for prior child RSA; conversely, within-dyad child-driven RSA synchrony was operationalized as the effect of fluctuations in a child’s RSA at one time point on subsequent fluctuations in their parents’ RSA, adjusting for prior parent RSA. Similar to a frequentist framework, if the 95% credible interval did not contain zero, the within-dyad interpersonal effect was determined to be non-null.

## Results

### Preliminary Analysis

#### Missingness

Missingness on RSA data was primarily due to either movement artifact or other noise that rendered the ECG data unusable or equipment error (e.g., loss of internet connection) that led to ECG data loss. Of the 28 families who participated in the study, two families (7.1%) had no usable parent RSA during the conflict discussion. In addition, one family (3.6%) who completed the conflict discussion task did not complete the positive event-planning task. Thus, the final N was 26 families during the conflict discussion, 27 families for the positive event-planning task, and 25 families for both tasks.

Of families with task ECG data, missingness during the task ranged from 0% to 30.4% (M = 3.3%). When a period of 3 or more beats in the ECG data needed correcting, data were considered missing and not used in analysis. The interpolation generated imputed IBI values for portions of missing data; however, we excluded the interpolated values for missing portions ≥ 10 s to maintain the precision of imputation. In turn, when there was a segment of missing data in the interpolated IBI series, the RSA values would be missing from 16 s before the segment until 16 s after the segment due to the tapering approach used.

#### Stationarity of RSA

Like many time series models, DSEMs assume stationarity (i.e., data are mean-reverting with no time-related trends, as well as constant variance, constant autocovariance, and constant lagged covariance). Prior to analysis, the time series of the outcome variables (caregivers’ and daughters’ RSA) for each dyad were evaluated to determine if each met mean-level and trend-level stationarity using the augmented Dickey-Fuller (ADF; Dickey & Fuller, 1979) test for stationarity. In both the single mean and trend models, a lag of 1 was specified. During the positive event-planning task, there was one dyad (3.7%) who did not meet stationarity for caregiver and child RSA. We evaluated M*plus*’ built-in safeguards for addressing mild violations of stationarity, which includes removing inadmissible values from posterior distributions (e.g., standardized autoregressive coefficients greater than 1). The model converged and the number of discarded iterations was very small (0.20% of all iterations), suggesting these safeguards were acceptable (Asparouhov, 2020). Thus, we included all available data in analyses.

### Primary Analyses

In each single-level DSEM, the intercepts, variances and covariance, and all autoregressive or AR(1) paths and cross-lagged paths in daughters’ and caregivers’ RSA were estimated. Supplementary Table 1 presents descriptive statistics and zero-order between-dyad correlations for the primary study variables.

#### Conflict Discussion Task

Unstandardized estimates of the cross-lagged regression path intercepts, aggregated across all single-level DSEMs, are described in the text and shown in Supplementary Table 2 and Supplementary Fig. 1a, b. Fifteen families exhibited non-null child-driven synchrony during the conflict discussion task: Nine families (34.6%) exhibited positive child-driven synchrony, such that changes in children’s RSA positively predicted changes in their parent’s subsequent RSA, whereas six families (23.1%) exhibited negative child-driven synchrony, such that changes in children’s RSA negatively predicted changes in their parent’s subsequent RSA. Fifteen families exhibited non-null parent-driven synchrony during the conflict discussion task: five families (19.2%) exhibited positive parent-driven synchrony, and ten families (38.5%) exhibited negative parent-driven synchrony.

#### Positive Event-Planning Task

Unstandardized estimates of the cross-lagged regression path intercepts, aggregated across all single-level DSEMs, are described in the text and shown in Supplementary Table 3 and Fig. 2a, b. Thirteen families exhibited non-null child-driven synchrony during the positive event-planning discussion task: seven families (25.9%) exhibited positive child-driven synchrony, such that changes in children’s RSA positively predicted changes in their parent’s subsequent RSA, whereas six families (22.2%) exhibited negative child-driven synchrony, such that changes in children’s RSA negatively predicted changes in their parent’s subsequent RSA. Eight families exhibited non-null parent-driven synchrony during the positive event-planning: three families (11.1%) exhibited positive parent-driven synchrony, and five families (18.5%) exhibited negative parent-driven synchrony.

#### Within-Dyad Similarity Across Tasks

Table 2 summarizes the presence and direction of parent- and child-driven RSA synchrony, per task. Cohen’s kappa evaluated whether dyads were likely to have similar patterns of non-nullness (either positive or negative) in child-driven and parent-driven synchrony across each interaction context. Kappas for dynamics were negative (see Table 2; range: -0.129–-0.279, all approximate *p*’s > 0.15), indicating that dyads were not more likely to exhibit the same pattern of synchrony during each task, and may even be more likely to show the opposite pattern of synchrony.

**Table 2:** Presence and direction of within-dyad parent- and child-driven RSA synchrony, per task

	Conflict Discussion	Positive event-planning discussion	Cohen’s kappa
Child-driven synchrony			
% Positive	34.6%	25.9%	-0.277
% Negative	23.1%	22.2%	-0.279
Parent-driven synchrony			
% Positive	19.2%	11.1%	-0.129
% Negative	38.5%	18.5%	-0.182

Positive synchrony refers to changes in RSA that were matched in direction. Negative synchrony refers to changes in RSA that were opposite in direction

## Discussion

Historically, RDoC has viewed the units of analysis in its matrix as trait-like dispositions (Patrick & Hajcak, 2016). However, the extant interpersonal dynamics literature demonstrates youth biobehavioral components exhibit state-like fluctuations, which are inextricably linked to unfolding changes in their caregivers’ biobehavioral functioning. Using rigorous methods to assess within-dyad synchrony, most (but not all) parent-daughter dyads exhibited interpersonal RSA synchrony (either parent- or child-driven) during a paradigm designed to activate negative valence systems (the conflict discussion task). Notably, parent-daughter synchrony was generally bidirectional, with opposing patterns of parent- versus daughter-driven RSA synchrony that may jointly contribute to maintaining dyadic equilibrium. Positive, in-phase child-driven synchrony may reflect parents’ cognitive and behavioral attunement with their children (Armstrong-Carter et al., 2021; Gao et al., 2023; Helm et al., 2018; McKillop & Connell, 2018), or alternatively, stress contagion in the context of heightened negative emotions or risk (Birk et al., 2022; Davis et al., 2018). In contrast, anti-phase parent-driven synchrony may arise from parents’ need to recruit their own physiological resources to “share the load” and regulate their child’s emotions and sustained engagement in the task (Davis et al., 2018). These preliminary results represent a descriptive characterization of between-dyad variation in within-dyad RSA synchrony that requires replication, extension, and exploration of intrapersonal and contextual correlates of these dynamics.

Results also support the need to assess caregiver-child dynamics using assessment paradigms sensitive to domain-specific alterations. Compared to the conflict discussion task, there was weaker evidence of parent-daughter synchrony during a paradigm designed to activate positive valence systems (the positive event-planning task). Further, there was null agreement or even divergence in within-dyad synchrony across paradigms; caregiver-child dynamics may vary within dyads and across interpersonal environmental contexts, influenced by the intersecting domains that are elicited. Taken together, these preliminary results suggest that parent- and child-led RSA synchrony varies *between* dyads and also *within* dyads across meaningfully separate paradigms. In the remainder of the discussion, we propose an agenda for advancing RDoC that builds on this preliminary work and discusses the clinical implications for dyadic assessment and intervention techniques.

### Our proposed approach for advancing RDoC

#### Leverage Assessment Paradigms to Evaluate Cross-Domain Intersections

Examining cross-domain intersections using multiple units of analysis and assessment paradigms adds to the limited work that examines dynamics across multiple contexts (DePasquale, 2020) and may offer greater explanatory power in uncovering differences in interpersonal dynamics than extant approaches. We argue for deepening the characterization of existing interpersonal assessment paradigms according to the processes that they elicit. For example, whereas prior work has found between-group differences in caregiver-child synchrony during challenging or non-challenging tasks (Birk et al., 2022; DePasquale, 2020; Di Lorenzo et al., 2022; Miller et al., 2023; Provenzi et al., 2018), we argue that existing paradigms can be understood as eliciting intersecting processes across different domains (e.g., conflict discussion tasks elicit social, cognitive, arousal/regulatory, and negative valence systems). The orthogonality of domains (and the constructs within domains), units of analysis, and assessment paradigms facilitates examination of cross-domain intersections within the RDoC framework and advances the study of interpersonal dynamics. One such example (shown in Table 1) is the cross-domain intersection between social processes (indexed by vagal functioning) and arousal and regulatory processes (elicited by HRV paradigms).

**Fig. 1:**
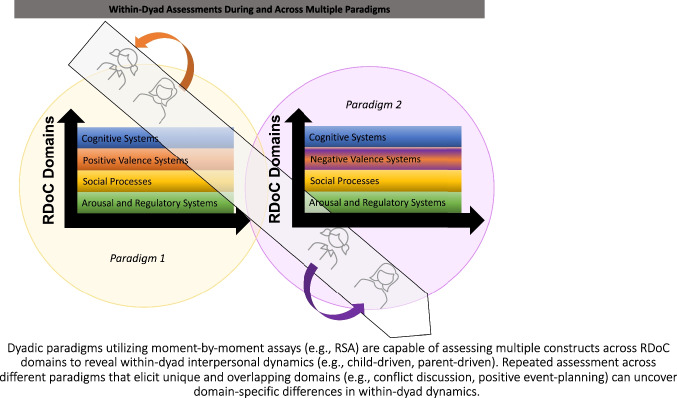
Reimagined RDoC matrix through the lens of interpersonal dynamics

Extending prior evidence that interpersonal dynamics are specific to the paradigm in which they are assessed (e.g., Armstrong-Carter et al., 2021), our results highlight distinct patterns of RSA dynamics across the conflict discussion and event-planning tasks. For example, caregiver-child RSA dynamics during discussion tasks reflect not only bidirectional influences between dyad member’s intersecting arousal and social processes (e.g., reflected in HF-HRV/RSA), but are also intertwined with cognitive processes (e.g., language, memory). However, as conflict discussion differs from pleasant event-planning in its activation of negative versus positive valence systems, differences in RSA dynamics elicited during these interpersonal assessment paradigms may reflect specific differences in affective processes. We contend that the *state-like* nature of interpersonal dynamics cannot be characterized without assessment of dynamics *during and across multiple* different salient interaction contexts; Fig. 1 represents a reimagined RDoC heuristic that highlights cross-domain intersections that unfold during and across diverse interpersonal assessment paradigms. In turn, assessment of dynamics in multiple contexts can help pinpoint specific domains of functioning in which dynamics may be altered. Importantly, not all dyads are equally sensitive to domain-specific (e.g., negative versus positive valence system, respectively) alterations, suggesting assessment paradigms may be directly translated into clinical practice to assess domain-specific alterations in dyadic functioning that may be health-promoting or maladaptive.

#### Move from the “What” to the “How” of Interpersonal Dynamics

Interpersonal dynamics are typically assessed with concurrent models that examine simultaneous linkage or matching of biobehavioral components. We encourage the use of multilevel time-lagged models that directly estimate the *drivers* of synchrony, or the unique effects of each partner on the other, over and above intrapersonal stability from one moment to the next (Armstrong-Carter et al., 2021; DePasquale, 2020; Helm et al., 2018; Miller et al., 2023). Within-dyad, time-lagged models (including DSEMs) strengthen conclusions about potentially causal interpersonal dynamics *between* the caregiver and the child, rather than demonstrating dyadic responses to shared external circumstances. Despite limitations to causal inference (e.g., specificity to time lag and interval; possible omitted time-varying confounds), identifying distinct child- and parent-driven processes informs understanding of *how* momentary fluctuations in individual-level RDoC units *may* be maintained or exacerbated over time through their immediate effects on the environment (i.e., on the other member of the dyad). This knowledge may lead to the identification of novel biomarkers and prevention/intervention targets that warrant subsequent investigation with experimental within-person designs (Hamaker et al., 2018).

We also acknowledge that theoretical and methodological innovations are needed to determine the appropriate temporal units for moment-to-moment assays. We examined second-by-second interpersonal parent–child dynamics, based on our prior theory-informed work on second-by-second dynamics in individual RSA and behavioral and mental health outcomes (Somers et al., 2021a, b). In contrast to prior work that has examined dynamics across wider (i.e., 15-to-30-s) epochs, examining second-by-second dynamics in RSA afforded the opportunity to examine RSA synchrony comparable to the time scale on which RSA-mediated influences on behavior are thought to occur (Somers et al., 2021b). In keeping with the majority of the RSA synchrony literature, we focused on within-dyad coordination in RSA over time; however, recent work has also begun to examine synchrony in second-by-second changes in RSA (Creavy et al., 2020; Ravindran et al., 2021), warranting comparison across alternative conceptualizations of RSA synchrony. There also may be unique patterns and mental health correlates of interpersonal dynamics when assayed at different timescales (e.g., Hollenstein et al., 2013; Zhang et al., 2022). We also echo Rohrer and Murayama (2023) in noting that there is not just *one* interpersonal dynamic, but rather multiple interpersonal dynamics operating at potentially different timescales during interactions and across development, and the timescale selected for a given study should be appropriate given the motivating theoretical framework. As an alternative to discrete time approaches where a lag must be specified, interpersonal dynamics can also be examined with continuous time approaches (e.g., multilevel ordinal differential equations) which yield parameters that are not specific to a particular time interval.

#### Link Alterations in Intrapersonal Functioning with Within-Dyad Processes

Moment-to-moment interpersonal dynamics are likely to constrain children’s neurodevelopment and emerging mental health problems and social competencies. At the same time, early-emerging mental health concerns may impact interpersonal dynamics in ways that exacerbate or perpetuate symptoms through transactions with the environment (Lougheed, 2020). Identifying intrapersonal correlates of maladaptive interpersonal dynamics could suggest novel, tractable endophenotypes that serve as early risk indicators and/or targets for interventions that seek to improve person-environment transactions and in turn prevent clinically significant impairment (Cuthbert, 2014). An intermediary step in this research agenda is to develop an archaeology of interpersonal dynamics, including assessment of how intrapersonal functioning in RDoC units may influence interpersonal dynamics across RDoC units. Although we focus on caregiver-child dynamics in units that can be assayed using moment-to-moment techniques, genes and molecules (e.g., oxytocin) are also relevant to interpersonal dynamics (e.g., Feldman et al., 2007, 2010; Markova & Nguyen, 2022). Heritable individual characteristics non-randomly elicit responses from the environment (i.e., evocative gene-environment correlation; Scarr & McCartney, 1983), such that child-led dynamics, or children’s influence on their caregivers, may in part reflect heritable child risk factors (e.g., Liu et al., 2020). Concurrent cross-unit associations also point to intrapersonal building blocks of caregiver-child dynamics during real-time interactions (e.g., evidence that greater conversational turn-taking is associated with more positive brain-based interpersonal dynamics; Ratliff et al., 2022).

Study design features could also be leveraged to improve traction on understanding between-person differences in within-dyad caregiver-child interpersonal dynamics. The active roles that children play in shaping their environments exist on two interdependent levels of analysis: (1) between-families, and (2) within families, during moment-to-moment interactions. These often-overlooked or uncovered child-driven influences may be more apparent when researchers: (a) recruit samples enriched for *child* transdiagnostic risk factors (e.g., negative emotionality); (b) minimize effects of potential confounders (e.g., child gender) through within-group designs; and (c) evaluate interpersonal dynamics during developmental transition periods (e.g., transition to adolescence) where these dynamics are hypothesized to undergo recalibration in light of developing biological, psychological, and social processes. Whereas prior work that failed to detect evidence of child-driven RSA synchrony across conflict discussion recruited families with adolescents, with a range of exposure to maternal depressive symptoms (e.g., McKillop & Connell, 2018), our sample of emotionally at-risk preadolescent girls and their caregivers may have optimized detection of child-driven RSA synchrony during conflict discussion. The broader environmental context (e.g., household socioeconomic resources, culture, neighborhood, etc.) may influence interpersonal dynamics directly (e.g., through influencing the likelihood of experiencing or expressing certain types of affect or behavior) or indirectly (e.g., through influencing appraisal of interaction goals); contextual factors may also alter the effects of interpersonal dynamics on health (Paley & Hajal, 2022). Future research using two-level DSEMs, with larger samples (e.g., 200 dyads with 100 or more time points; Schultzberg & Muthén, 2018), are needed to examine within-dyad synchrony across units of analysis and between-dyad differences in synchrony due to child and parent emotional risk, gender, age, and contextual risk due to socioeconomic disadvantage or ethnic minority status. Longitudinal designs (e.g., pre-post DSEM; measurement burst designs) are also needed to evaluate stability and change in interpersonal dynamics and their correlates in the context of specific developmental challenges and transdiagnostic risk factors (Davis et al., 2018; DePasquale, 2020).

### Clinical implications

A translational approach requires accumulating knowledge of disrupted interpersonal dynamics implicated in youth psychological distress and integrating these findings into clinical practice. Clinical assessment techniques that leverage the interpersonal context of child psychopathology should be informed by evidence (including that provided by our proof-of-concept study) that these dynamics are state-like properties of the dyad *and* the interaction context in which they are elicited. In addition to informing development of new diagnostic indexes, interpersonal dynamics that reinforce or exacerbate child psychopathology through transactions with the caregiving environment may also be prognostic indicators of worsening symptom course or (individually-focused) treatment response (De Rubeis & Granic, 2012). Dyad-oriented assessment and treatment strategies may mitigate stigma associated with treatment-seeking for parenting support, while also underscoring each individual’s agency within the social environment. Assessing interpersonal dynamics in multiple interaction contexts that differentially elicit key domains of functioning may also help families identify strengths as well as areas for growth.

The study of interpersonal dynamics also points to family-centered intervention strategies for multiple youth risk or clinical conditions. Interventions that leverage dynamic caregiver-child interactions have traditionally focused on families with infants and younger children (e.g., infant-parent psychotherapy); however, the extant literature, including the present findings, underscores how the interpersonal context remains salient across the lifespan. Empirically-supported interventions that facilitate adaptive bidirectional dynamics, including both parent-led dynamics (e.g., teaching parents constructive emotion socialization skills, such as modeling coping strategies) and child-led dynamics (e.g., teaching parents to be aware of their own responses in the face of child distress), are needed for families with youth of all ages. Practitioners could use dyadic neurophysiological feedback, along with well-validated video feedback techniques (Balldin et al., 2018; Fukkink, 2008), to tailor feedback for parents and children about their interpersonal dynamics. Dyadic feedback could be used as an initial assessment strategy and intervention procedure; with repeated feedback over time, parents and children may respond to each other more contingently and ultimately adaptively (Ratliff et al., 2022). Outside of therapy, other reflective practices (e.g., mindful awareness, Feelings Thermometer) could be used to help inhibit prepotent responses, thus leading to new, adaptive interpersonal dynamics (Hajal & Paley, 2020).

## Conclusions

To advance innovation in clinical assessment and intervention, a rigorous understanding of biobehavioral and environmental mechanisms of child psychopathology is needed. The RDoC matrix provides a guiding framework for this goal but traditional *intrapersonal* assessment of its units of analysis fails to consider critical, real-time *interpersonal* influences on caregivers’ and children’s functioning. Compelling, well-replicated evidence of caregiver-child interpersonal dynamics in each unit of analysis illuminates that the interpersonal environment is *already* intrinsic to the RDoC matrix. Studying each unit of analysis through the lens of interpersonal dynamics makes explicit this intrinsic connection between intrapersonal functioning and moment-to-moment environmental influences during social interactions. Further, we argue that the RDoC framework and the study of interpersonal dynamics can be deepened through the examination of cross-domain intersections, which involve interrelated domains elicited by units of analysis and assessment paradigms, and evaluation of the drivers of interpersonal dynamics. Our methodological proof-of-concept among a sample of emotionally at-risk preadolescent girls and their parents offers empirical support for the proposed research agenda. Clinically, assessment and intervention techniques that leverage the caregiver-child interpersonal context may bring light to novel approaches by to fulfill the promises of the RDoC framework for improving child and adolescent mental health.

### Supplementary Information

Below is the link to the electronic supplementary material.

Supplementary file1 (DOCX 69 KB)

## Data Availability

The data and code that support the findings of this study are available from the corresponding author upon reasonable request.
