# Supplementary material for: Integrating Biobehavioral and Environmental Components of Developmental Psychopathology via Interpersonal Dynamics: An RDoC-Advancing Model
Source: Res Child Adolesc Psychopathol. 2023 Aug 21;52(4):491–504. doi: 10.1007/s10802-023-01110-8 (PMC10879449; doi:10.1007/s10802-023-01110-8)
Supplement: Supplementary file 1 — Supplementary file1 (DOCX 69 KB) [file 10802_2023_1110_MOESM1_ESM.docx]

*Supplementary Table 1*. Between-dyad descriptive statistics and Pearson correlations in primary variables

|  | M | SD | 1 | 2 | 3 |
| --- | --- | --- | --- | --- | --- |
| 1. Caregiver Mean RSA (Conflict) | 6.02 | 0.91 | --- |  |  |
| 2. Caregiver Mean RSA (Positive Event) | 6.24 | 1.32 | .51** | --- |  |
| 3. Daughter Mean RSA (Conflict) | 5.43 | 0.87 | .45* | .58** | --- |
| 4. Daughter Mean RSA (Positive Event) | 5.91 | 1.65 | .25 | .32 | .49* |

*Note*. RSA = respiratory sinus arrhythmia. Conflict = Conflict discussion. Positive Event = Positive event-planning interaction.

** *p* < .01. * *p* < .05.

*Supplementary Table 2*. Presence and direction of within-dyad parent- and child-driven RSA synchrony, in the conflict discussion task

|  | Positive Parent-Driven | Null Parent-Driven | Negative Parent-Driven |
| --- | --- | --- | --- |
| Positive Child-Driven | 0 | 1 | 8 |
| Null Child-Driven | 0 | 9 | 2 |
| Negative Child-Driven | 5 | 1 | 0 |

*Note.* Positive synchrony refers to changes in RSA that were matched in direction. Negative synchrony refers to changes in RSA that were opposite in direction.

*Supplementary Table 3*. Presence and direction of within-dyad parent- and child-driven RSA synchrony, in the positive event-planning task

|  | Positive Parent-Driven | Null Parent-Driven | Negative Parent-Driven |
| --- | --- | --- | --- |
| Positive Child-Driven | 0 | 4 | 3 |
| Null Child-Driven | 0 | 12 | 2 |
| Negative Child-Driven | 3 | 3 | 0 |

*Note.* Positive synchrony refers to changes in RSA that were matched in direction. Negative synchrony refers to changes in RSA that were opposite in direction.

*Supplementary Figure 1a.* Forest plot of child-driven synchrony during the conflict discussion task


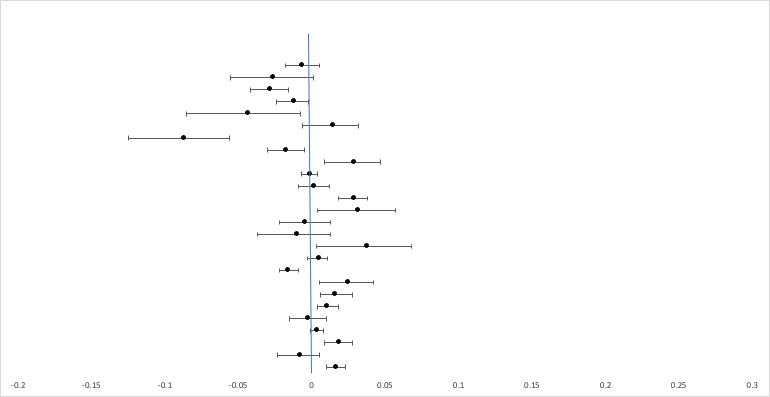


*Note*. 95% credible intervals (CrI) are shown for each dyad. 95% CrI that do not contain 0 are considered non-null.

*Supplementary Figure 1b*. Forest plot of parent-driven synchrony during the conflict discussion task


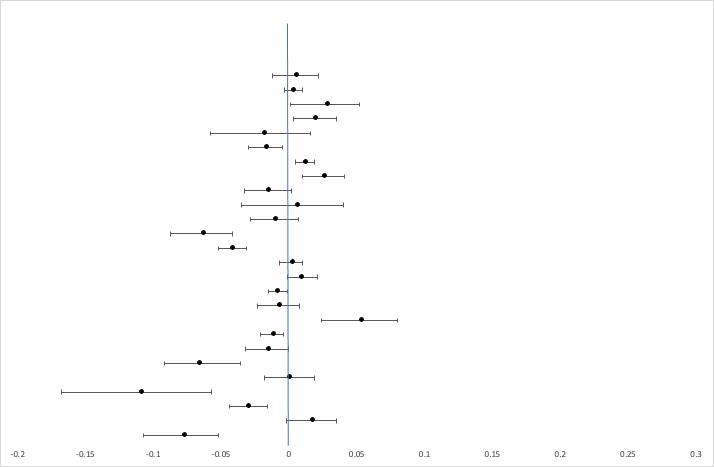


*Note*. 95% credible intervals (CrI) are shown for each dyad. 95% CrI that do not contain 0 are considered non-null.

*Supplementary* *Figure 2a*. Forest plot of child-driven synchrony during the positive event-planning task


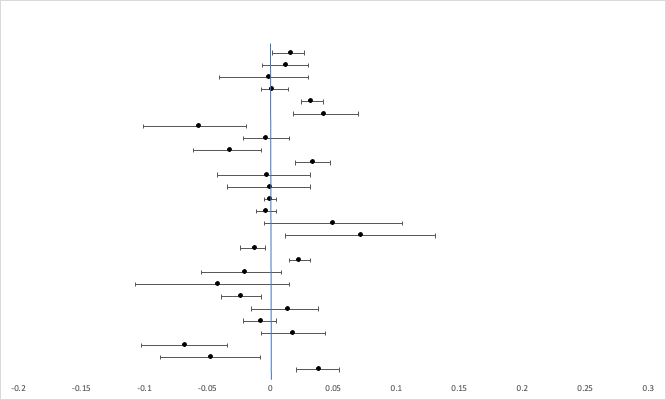


*Note*. 95% credible intervals (CrI) are shown for each dyad. 95% CrI that do not contain 0 are considered non-null.

*Supplementary Figure 2b*. Forest plot of parent-driven synchrony during the positive event-planning task


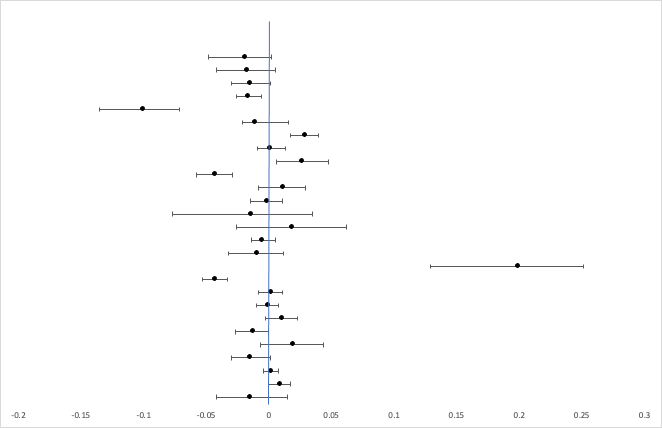


*Note*. 95% credible intervals (CrI) are shown for each dyad. 95% CrI that do not contain 0 are considered non-null.
